# The effect of combining different sampling tools on the performance of electromagnetic navigational bronchoscopy for the evaluation of peripheral lung lesions and factors associated with its diagnostic yield

**DOI:** 10.1186/s12890-023-02711-1

**Published:** 2023-11-08

**Authors:** Javier Flandes, Francisco B. Martinez-Muñiz, Juan José Cruz-Rueda, Francisco J. Soto, Adnan Majid, Eduardo Tuta-Quintero, Luis F. Giraldo-Cadavid

**Affiliations:** 1 Chief of Bronchology and Interventional Pulmonology Unit, IIS-Fundación Jiménez Díaz, CIBERES, Avenida Reyes Catolicos No 2, 28040 Madrid, Spain; 2 Department of Pulmonology, Hospital de La Línea de La Concepción, Cádiz, Spain; 3 Department of Pulmonology, Hospital Universitario Torrecárdenas, Almería, Spain; 4 Division of Pulmonary and Critical Care Medicine, Department of Medicine, University of Tennessee Graduate School of Medicine, Knoxville, TN USA; 5 Division of Thoracic Surgery and Interventional Pulmonology, Department of Surgery, Beth Israel Deaconess Medical Center, Harvard Medical School, Boston, MA USA; 6 Master’s Candidate in Epidemiology, Universidad de La Sabana, Chía, Colombia; 7 Professor of Medicine at Facultad de Medicina, Autonorte de Bogota. Chía, Universidad de La Sabana. Address: Universidad de La Sabana, Km 7, 250001 Cundinamarca, Colombia; 8 Chief of the Interventional Pulmonology Service at Fundacion Neumologica Colombiana, Cra. 13B#161 - 85, 110131 Bogotá, Colombia

**Keywords:** Electromagnetic navigational bronchoscopy, Solitary pulmonary nodules, Peripheral lung lesions

## Abstract

**Background:**

We assessed the performance of Electromagnetic navigational bronchoscopy (ENB) as a standalone diagnostic technique and the performance of different sampling tools used during the procedure.

**Methods:**

We recruited 160 consecutive patients who underwent ENB for peripheral lung lesions (PLL) at a tertiary care centre. The diagnostic performance of ENB and sampling tools was assessed using a logistic regression model and a ROC-curve in which the dependent variable was diagnostic success. A multivariate model was built to predict diagnostic success before performing ENB to select the best candidates for the procedure.

**Results:**

Most patients with PLLs in the study were male (65%), with a mean age of 67.9 years. The yield was 66% when the most common techniques were used together as suction catheter + transbronchial biopsy forceps (TBBx) + bronchoalveolar lavage + bronchial washing (*p* < 0.001) and increased to 69% when transbronchial needle aspiration (TBNA) and cytology brush were added (*p* < 0.001). Adding diagnostic techniques such as TBBx and TBNA resulted in an increase in diagnostic performance, with a statistically significant trend (*p* = 0.002). The logistic model area-under the ROC-curve for diagnostic success during ENB was 0.83 (95% CI:0.75–0.90; *p* < 0.001), and a logit value ≥ 0.12 was associated with ≥ 50% probability of diagnostic success.

**Conclusions:**

ENB, as a stand-alone diagnostic tool for the evaluation of PLLs when performed by experienced operators using a multi-modality technique, has a good diagnostic yield. The probability of having a diagnostic ENB could be assessed using the proposed model.

**Supplementary Information:**

The online version contains supplementary material available at 10.1186/s12890-023-02711-1.

## Introduction

Lung cancer screening programmes and the widespread availability of computed tomography (CT) of the chest have increased the detection of solitary pulmonary nodules [1–3]. Tools such as positron emission tomography/computed tomography (PET/CT) evaluation have decreased the need for unnecessary solitary pulmonary nodules resection [4]. Nevertheless, it has diagnostic limitations and might not be universally available [5]. The yield of conventional bronchoscopy in peripheral lung lesions (PLLs) is limited [6], as low as 14% for those in the outer periphery [7, 8]. Electromagnetic navigational bronchoscopy (ENB) provides a safe and higher-yield diagnostic procedure for the evaluation of PLLs [9–12]. Its yield can be significantly affected by the simultaneous use of other tools such as radial endobronchial ultrasound (r-EBUS), fluoroscopy, rapid on-site evaluation (ROSE), or whether the procedure is done under moderate sedation vs. general anesthesia [13, 14]. With over 30 studies reporting on endoscopic ENB yield, most of them have used one or more of the above supporting diagnostic tools and techniques [6, 10, 12–44]. Therefore, data on the stand-alone diagnostic performance of ENB are limited [39, 45, 46]. We sought to identify the performance of ENB as a stand-alone diagnostic technique and the effect of the various sampling techniques used during the procedure.

## Methods

Observational analytical single-center study performed in a prospective institutional registry of patients with peripheral pulmonary lesions of the Fundación Jiménez Díaz University Hospital in Madrid, attached to the Autonomous University of Madrid with patients who were recruited to be included in the NAVIGATE study [10]. The primary goals of the study included the identification of variables predicting the yield of ENB as a stand-alone diagnostic technique. Secondary aims included ENB yield after an 18-month follow-up period, yield of individual tissue-sampling tools, and pneumothorax rate.

### Eligibility criteria

The eligibility criteria included consecutive patients who underwent ENB for the work-up of PLLs and who had clinical and radiological data available during an 18-month follow-up period (spanning from July 2011 to October 2015). Exclusion criteria included difficulties tolerating moderate sedation, evidence of a visible endobronchial lesion, or a different ENB indication, such as fiducial marker placement.

### Clinical variables

Demographics, smoking history, presence of chronic obstructive pulmonary disease (COPD) [47, 48], COPD severity [47], and previous lung metastasis from extrathoracic primary (extrathoracic cancer). Never-smokers and those who had quit for > 15 years were labelled as “non-smokers”. Nodule characteristics recorded included size in its three spatial axes [x, y, z], largest diameter on any axis [49], lobar location [16], fissure attachment (fissure adherence involving at least 1/3 of the lesion), pleural attachment, distance to the pleura, and bronchus sign [50]. A PET/CT standardized uptake value (SUV) of ≥ 3 was considered suspicious for malignancy.

### ENB System

The system included the superDimension™ navigation system software version 6.0 (Medtronic, Minneapolis, MN), the Edge™ locatable guide, and the Edge™ 180° degree firm extended working channel. A therapeutic, flexible video bronchoscope with a 2.8-mm working channel was used in all procedures (Olympus; Tokyo, Japan).

### Procedure and sample processing

ENB was performed in a standard fashion following the manufacturer’s protocol [51]. The lesions in which ENB was not diagnostic underwent chest CT-guided biopsy, endobronchial ultrasound (EBUS), or thoracic surgery.

ENB samples that yielded specific diagnoses (both benign and malignant), corroborated by surgical biopsy/resection results, clinical and imaging follow-up, or, in the case of benign disease, clinical and imaging assessments, were classified as diagnostic ENB. In contrast, ENB was categorized as non-diagnostic when it failed to provide a specific diagnosis. In cases of non-diagnostic ENB, we determined the final diagnosis through alternative procedures, including thoracic surgery, CT-guided biopsy, or linear EBUS (EBUS-TBNA). For patients who underwent surgery following a diagnostic ENB sample (i.e., therapeutic lung tumour resection), we based the final diagnosis on the histological examination of the surgical specimen. Additionally, we followed up patients for at least 18 months, during which clinical and imaging examinations were conducted to evaluate the consistency or discrepancy with the ENB results.

All patients had chest CT scan images obtained on Digital Images and Communications in Medicine format with a 512 × 512 resolution, a slice thickness of 1 mm, and an overlap of 0.8 mm. Images were uploaded using iLogic® software to create a three-dimensional road map. Sedation and monitoring during bronchoscopy were conducted according to the recommendations of the Spanish Society of Pulmonology and Thoracic Surgery [52] and the American College of Chest Physicians [53]. Topical anaesthesia was provided with lidocaine. Intravenous midazolam (median dose, 4.5 mg) and fentanyl (median dose, 100 µg) were used for moderate sedation. General anaesthesia, fluoroscopy, ROSE, or r-EBUS were not used during any ENB procedure.

Tools and techniques used during ENB included: suction catheter [24], transbronchial biopsy forceps (TBBx), cytology brush (CB), transbronchial needle aspiration (TBNA), bronchoalveolar lavage (BAL), and bronchial washing. Supplementary Table 1 provides individual technique details.

### Sample size

We estimated that the study would require a minimum sample size of 160 patients to have a least 80 patients with the outcome (diagnostic ENB) in the more demanding scenario of 50% of patients with diagnostic ENB [54, 55]. These 80 patients would be sufficient to build a binary logistic regression multivariate model with 8 covariates (10 patients with the outcome per covariates).

### Statistical analysis

Statistical analysis was performed using STATA version 17 software (STATA Corp., Texas, USA). Descriptive statistics for all continuous variables were summarised as means, standard deviations (SD), medians, and interquartile ranges. Frequency distributions and percentages were reported for discrete variables. The association between each variable and the diagnostic yield was analysed. For each outcome, associations with the corresponding set of variables were checked by χ^2^ or Fisher’s exact test (for categorical variables). Confidence intervals (CI), odds ratios (OR), and p-values were reported; two-tailed p values of less than 0.05 were considered to indicate statistical significance. A receiver operating characteristic (ROC) curve was plotted, and the area under the ROC curve was calculated. We evaluated the statistical significance of the trend in the use of combined techniques using the Cochran–Armitage statistical test.

## Results

### Demographic characteristics and key results

ENB was performed on 173 patients, but 13 patients were excluded according to eligibility criteria. The mean age was 67.9 years (SD:11), and 65% were male (104/160) (Table 1). While we did not collect specific procedure time data for individual patients, it is worth noting that, on average, each ENB procedure at our institution typically lasts approximately 90 min. The overall diagnostic yield was 69.4%, based on 18-month follow-up data. Sensitivity, specificity, negative predictive value (NPV), and positive predictive value (PPV) were 59%, 100%, 45.6%, and 100%, respectively.

**Table 1:** Demographics, Lesions and Procedure Characteristics

Number of patients n(%)	160 (100)
Mean age, years x(SD)	67.9 (11)
Older than 75 years n(%)	110 (68.7)
Under than 75 years n(%)	50 (31.2)
Gender, Male n(%)	104 (65.0)
Current or former smoker^a^n(%)	70 (43.7)
COPD diagnosis^b^n(%)	67 (51.9)
Previos extrathoracic cancer n(%)	51 (31.8)
Mean nodule diameter mm (IQR)	16 mm (11.5–21.5)
Nodule location n(%)	
Upper lobes	93 (58.1)
Middle lobe	15 (9.3)
Lower lobes	52 (32.5)
Nodule uptake on PET-CT^c^n(%)	
< 3 SUV	26 (16.2)
≥ 3 SUV	103 (64.3)
Distance to pleura n(%)	
< 10 mm	90 (56.2)
≥ 10 mm	70 (43.8)
Bronchus sign presence n(%)	88 (55.0)
Perifissural lesion n(%)	34 (21.2)
Overall ENB diagnosis yield, n(%)	111 (69.4)
Malignant etiology of PLL, n(%)	118 (74)

*n* Number, *SD* Standard deviation, *m* Median, *SUV* Standardized uptake value, *ENB* Electromagnetic navigational bronchoscopy, *PLL* Peripheral lung lesions

^a^Former smokers were those having quitted smoking in the last 15 years

^b^Spirometry available for 129 patients

^c^PET-CT available for 129 patients

### Univariate analysis of diagnostic yield

**Fig. 1:**
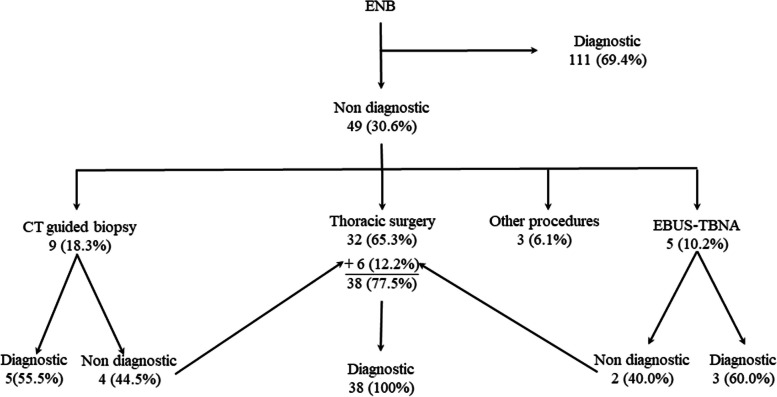
Diagnostic algorithm based on 18-month follow-up data. Notes: *ENB* Electromagnetic navigational bronchoscopy, *CT* Computer tomography, *EBUS* Endobronchial ultrasound, *EBUS-TBNA* Linear EBUS

ENB yielded a diagnostic result in 111 out of 160 cases (69.4%). In the remaining 49 cases with non-diagnostic ENB, the diagnosis was established through thoracic surgery in 38 cases (77.5%), CT-guided biopsy in 5 cases (10.2%), Linear EBUS (EBUS-TBNA) in 3 cases (6.1%), and other procedures in 3 cases (6.1%) (Fig. 1).

The diagnostic yields in patients with and without a history of extrathoracic cancer were 32% and 68%, respectively (*p* = 0.001) (Table 2). In patients with perifissural lesions, the yield decreased 17% compared to patients without these lesions (56% vs. 73%; *p* = 0.054), and lesions located in the inferior lobes presented a diagnostic yield of 65%. 74.4% (119/160) received a diagnosis of malignant tumour, 23.1% of benign tumour (37/160) and 2.5% of infections (4/160). The diagnostic yield of malignant tumours of primary pulmonary origin was 59%, metastatic lesions of extrathoracic origin 22%, and benign tumours 100%.

**Table 2:** Bivariate analysis of Diagnostic yield ENB based on demographic variables, lesion characteristic and etiology

Variables	No	Diagnostic yield	*p*-value*
**Age, > 75 years n(%)**			
No	110	67%	0.392
Yes	50	74%	
**Smoking history**			
No	90	63%	0.063
Yes	70	78%	
**History of extrathoracic cancer**			
No	109	68%	0.001
Yes	51	32%	
COPD diagnosis*			
No	62	61%	0.380
Yes	67	68%	
**COPD severity**			
Group I	9	60%	0.918
Group II	43	67%	
Group III	11	73%	
Group IV	4	75%	
**Location**			
Inferior lobes	52	65%	0.447
Non-inferior lobes	108	71%	
**Largest axis**			
< 15 mm	62	63%	0.158
≥ 15 mm	98	73.5%	
< 20 mm	109	70%	0.888
≥ 20 mm	51	69%	
**X axis**			
< 15 mm	85	67%	0.499
≥ 15 mm	75	72%	
**Y axis**			
< 15 mm	98	67%	0.484
≥ 15 mm	62	72.6%	
**Z axis**			
< 15 mm	91	64%	0.076
≥ 15 mm	69	77%	
**Perifissural lesion**			
No	126	73%	0.054
Yes	34	56%	
**Cavitation**			
No	148	69.6%	0.832
Yes	12	67%	
**Distance to pleura**			
< 10 mm	90	69%	0.880
≥ 10 mm	70	74%	
< 20 mm	116	71%	0.558
≥ 20 mm	44	66%	
**Pleural tail**			
No	108	66.7%	0.978
Yes	52	73%	
**Bronchus sign**			
No	72	60%	0.017
Yes	88	77%	
**PET/CT**^a^			
< 3SUV	26	69%	0.688
≥ 3 SUV	103	66%	
**Diagnosis confirmed by ENB and follow-up**			
**Malignant diseases**	119	59%	< 0.001
NSCLC + SCLC + Metastasis^b^	119	59%	< 0.001
Metastasis^b^	18	22%	
**Benign diseases**	**41**		
**Benign non-infectious diseases**	**37**	**100%**	** < 0.001**
Non-specific inflammatory nodules	31		
Granuloma	4		
COP	1		
Hamartoma	1		
**Infectious**	**4**	**100%**	**0.149**
Tuberculosis	3		
Aspergillosis	1		

*COPD* Chronic obstructive pulmonary disease, *PET/CT* Positron emission tomography/computed tomography, *SUV* Standardized uptake value, *NSCLC* Non-small cell lung cancer, *SCLC* Small-cell lung cancer, *COP* Cryptogenic organizing pneumonia

^*^The diagnosis of COPD was established in accordance with the GOLD spirometry criteria. Due to the availability of spirometry data for only 129 patients, classification regarding COPD status was not feasible for the remaining 31 patients

^a^PET-CT available for 129 patients

^b^Metastatic pathology in ENB and confirmed in the diagnostic follow-up. Metastasis are included in the count of malignant diseases (“NSCLC + SCLC + Metastasis”) and in the count of Metastasis, therefore, to determine the total count of both malignant and benign diseases accurately, one should sum the subtotal of malignant diseases (119) and the subtotal of benign diseases (41) while avoiding the duplication of metastatic cases. Non-specific inflammatory nodules and granulomas represent inflammatory and granulomatous lesions for which an infectious agent was not isolated, and these lesions either resolved or decreased in size during follow-up

### Multivariate analysis of factors affecting diagnostic yields

**Fig. 2:**
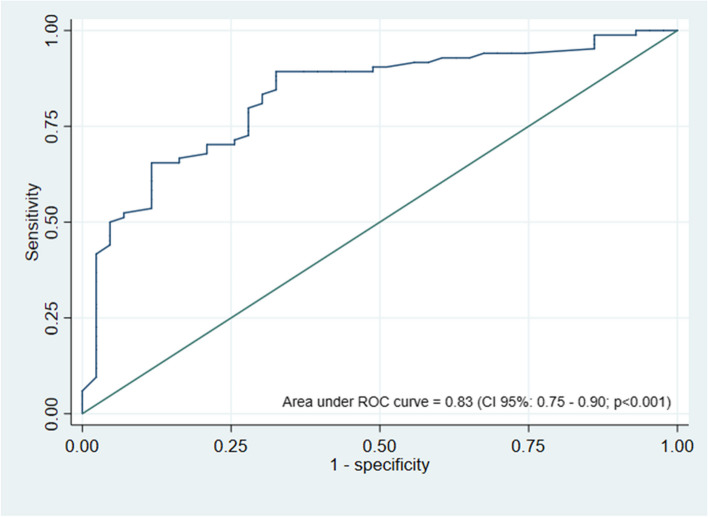
ROC curve of the predictive model for diagnostic success during ENB including the area under ROC curve

We identified several factors increasing the diagnostic yield, including lesion size of ≥ 15 mm in the Z axis, presence of bronchus sign, smoking history, and age > 75. On the other hand, the yield was decreased by factors like history of extrathoracic malignancy, perifissural lesions and location in lower lobes (Table 3). A prediction equation was developed using these variables (Table 4). The area under the ROC curve of the predictive model for diagnostic success during ENB was 0.83 (95% CI: 0.75–0.90; *p *< 0.001) (Fig. 2). We found that when the PLLs were identified as metastatic, the diagnosis decreased the ENB yield by 85%, with an OR of 0.15 (95% CI: 0.03–0.54; *p* = 0.01). The malignancy rate was higher for SU ≥ 3 (Supplementary Table 2).

**Table 3:** Multivariate analysis factors affecting diagnostic yield

Variables	Coefficient	OR	(95% CI)	*p*-value
Constant (K)				
History of extrathoracic cancer	-1.156	0.315	(0.119–0.796)	0.014
Perifissural lesion	-1.594	0.203	(0.063–0.604)	0.004
Smoking history	1.947	7.006	(2.536–21.96)	< 0.001
Age (≥ 75)	1.580	4.853	(1.655–16.16)	0.003
Z axis mm (≥ 15)	1.574	4.828	(1.762–14.81)	0.002
PET/CT SUV (≥ 3)	-2.377	0.093	(0.020–0.365)	< 0.001
Bronchus sign	1.416	4.120	(1.648–11.12)	0.002
Location (lower lobes)	-1.447	0.235	(0.069–0.741)	0.013

*PET/CT* Positron emission tomography/computed tomography, *SUV* Standardized uptake value

**Table 4:** ENB yield prediction equation

X*	Variables	Presence of	Absence of
X1	History of extrathoracic malignancy	1	0
X2	Perifissural lesion	1	0
X3	SUV ≥ 3 on PET/CT	1	0
X4	Lower lobe nodule location	1	0
X5	Age ≥ 75 years	1	0
X6	Smoking history (current or within 15 years)	1	0
X7	≥ 15 mm nodule diameter on Z axis	1	0
X8	Presence of bronchus sign	1	0

Prediction equation: $$\mathrm{Logit}=(-1.12*\mathrm{X}1)+(-1.59*\mathrm{X}2)+(-2.19*\mathrm{X}3)+(-1.34*\mathrm{X}4)+(1.30*\mathrm{X}5)+(1.85*\mathrm{X}6)+(1.45*\mathrm{X}7)+(1.39*\mathrm{X}8)+(1.25)$$

*ENB* Electromagnetic navigational bronchoscopy, *PET/CT* Positron emission tomography/computed tomography, *SUV* Standardized uptake value.*Model goodness of fit by Hosmer–Lemeshow test

### ENB Diagnosis at 18-month follow-up

The diagnostic yield of ENB was 69.4% (111/160). Of the patients who obtained a false negative by means of ENB, 100% (49/49) were eventually diagnosed with a malignant tumour. By contrast, in the patients whose diagnosis was confirmed, they presented a non-malignant diagnosis of 36.9% (41/111) and a malignant diagnosis of 63.0% (70/111). The diagnostic algorithm after an 18-month follow-up is shown in Fig. 1.

### Yield of bronchoscopic sampling techniques

**Fig. 3:**
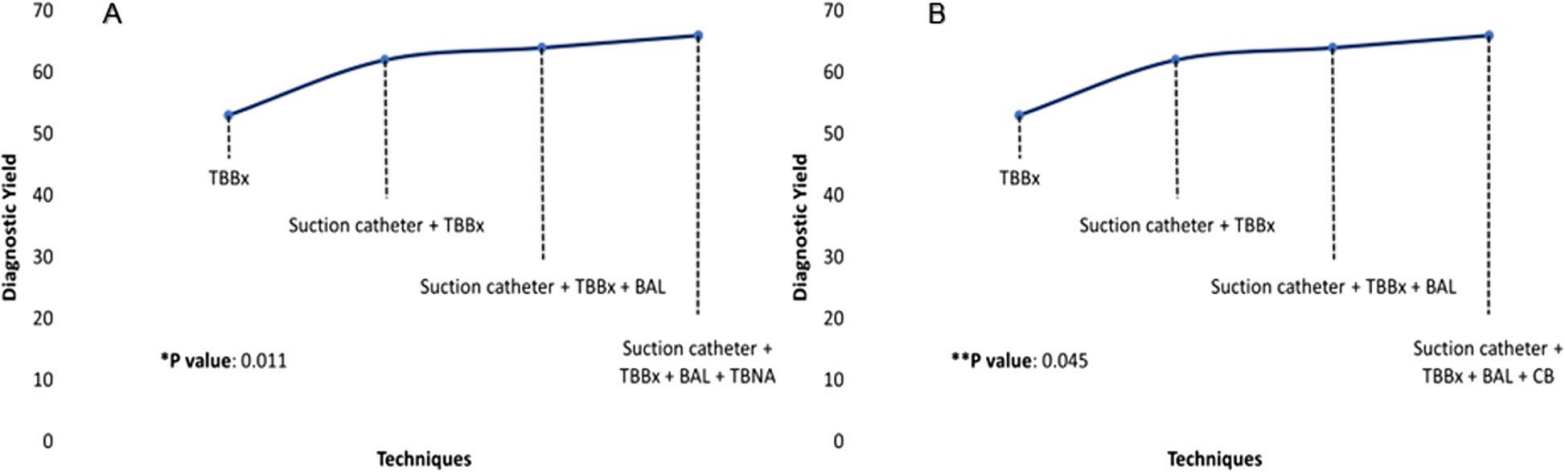
Diagnostic yield of the TBNA and CB with others sampling techniques. Notes: *TBBx* Transbronchial biopsies, *BAL* Bronchoalveolar lavage, *CB* Cytology brush, *TBNA* Transbronchial needle aspiration. *trend in the use of transbronchial needle aspiration with other combined techniques (Cochran–Armitage statistical test). ** trend in the use of cytology brush with other combined techniques (Cochran–Armitage statistical test)

CB and TBBx provided the highest yields, 51% and 53%, respectively (Table 5). Bronchial washing had the lowest yield (30%). The yield was around 66% when the suction catheter, TBBx, BAL, and CB were combined. Adding diagnostic techniques such as TBBx and TBNA increased the diagnostic performance and showed a statistically significant trend (*p* = 0.011; *p* = 0.045 and *p *= 0.002) (Fig. 3a and supplementary Fig. 1). Additionally, the combined use of TBNA or CB with other diagnostic techniques showed a significant increase in performance, with a significant trend (Fig. 3b).

**Table 5:** Diagnostic yield of techniques and tools used during ENB

	Total No	Diagnostic yield	Value *p*
Bronchial washing	155	30%	< 0.001
TBNA	51	43%	0.006
BAL	140	48%	< 0.001
Suction catheter	142	49%	< 0.001
CB	118	51%	< 0.001
TBBx	153	53%	< 0.001
Suction catheter + TBBx	136	62%	< 0.001
Suction catheter + TBBx + BAL	126	64%	< 0.001
Suction catheter + TBBx + CB	104	64%	< 0.001
Suction catheter + TBBx + BAL + CB	98	66%	< 0.001
Suction catheter + TBBx + BAL + CB + Bronchial washing	96	66%	< 0.001
Suction catheter + TBBx + BAL + TBNA	43	67%	< 0.001
Suction catheter + TBBx + BAL + CB + TBNA	39	68%	< 0.001
Suction catheter + TBBx + BAL + CB + TBNA + Bronchial washing	39	69%	< 0.001

*ENB* Electromagnetic navigational bronchoscopy, *TBBx* Transbronchial biopsies, *CB* cytology brush, *TBNA* Transbronchial aspiration, *BAL* Bronchoalveolar lavage

### Safety and pneumothorax

Pneumothorax occurred in 7.5% (12/160) of the procedures, but only 2.5% (4/120) required drainage. In total, 51 TBNA were performed, of which 7.8% (4/51) had pneumothorax as a complication. There was no statistical association between pneumothorax and the sampling tool used (Supplementary Table 3). No individual factor increased the pneumothorax risk.

## Discussion

We found a good diagnostic yield based on 18-month follow-up data and using ENB as a stand-alone diagnostic tool. This number is in the range of reported pooled yields (58.6%–84%) [6, 23, 29, 33, 35, 39, 40, 42, 45, 46, 56–58], it represents an average assessment of ENB. Our diagnostic yield is good when taking into account that our mean nodule diameter was smaller than those described in diagnostic ENB studies (Supplementary Table 4) [6, 23, 29, 33, 35, 39, 40, 42, 45, 46, 56–58].

Several studies are similar to ours, since they used only ENB for all of their cases [42, 45, 56–58]. For example, Bertoletti showed a yield of 77.4% but with a much larger nodule diameter (31.2 vs. 16 mm) [45]. Ozgul et al. examined ENB yield in 56 cases, but r-EBUS was used in 26 of those cases [39]. The yield for non-r-EBUS cases was 71.4%, which is similar to our study. Further, Raval and Amir reported an 83.3% yield with a relatively small lesion size (19.3 mm) [46]. Although only ENB was used, they utilised a tidal volume expiration mapped ENB (Veran Medical Technologies), which limits the comparison.

Some of our independent yield predictors have been previously identified in studies with multivariate analysis [14, 16, 18, 20, 22, 27, 49]. For example, smoking history increased our yield by sevenfold. Ost et al. also reported a smoking association, less strong [14]. The effect of smoking might be related to the localised distortion effect that it generates at the bronchial architecture level, triggered by the chronic inflammatory effect [47]. This could facilitate locatable guide access to nearby solitary pulmonary nodules. The presence of a bronchus sign on CT increased the yield by fourfold. Seijo et al. reported such an association with an eightfold increase in yield [42]. Our study confirms their findings, as a bronchus sign increased performance by 17% compared to patients without bronchial signs in our sample. Since bronchus sign was only present in about 50% of the patients, for cases without bronchus sign, it is our practice to make strategies, such as modifying the location of the locatable guidewire on at least two occasions, while obtaining transbronchial biopsies to avoid the “all-or-none” diagnostic phenomenon [14]. On this wise, samples are collected from various regions close to the lesion. We have yet to verify this fact, but we believe that it could mitigate the effect of not presenting the bronchus sign.

PLLs in the lower lobes decreased our yield by close to 80% [16, 22]. This could be explained by diaphragmatic movement during inspiration, with a difference in PLLs location of up to 2.5 cm. [22, 59] Lesion diameter of > 20 mm in its largest axis [14, 49] and > 30 mm^18^ correlated with higher diagnostic yield in other studies. For us, a lesion size of ≥ 15 mm in the Z axis increased the yield close to fivefold. We postulate that a larger lesion on the Z-axis might provide better endobronchial exposure. This requires additional validation. Perifissural lesions decreased the diagnostic yield; this might occur because of a reduction in bronchus size and a more tortuous airway pattern, limiting the advance of the locatable guide. Age of ≥ 75 increased our ENB yield by 4.8-fold. The rationale for this effect is unclear.

One of the clinical factors that negatively modify diagnostic yield is presenting a personal history of previous extrathoracic cancer before ENB [60]. Those patients had a higher percentage of metastatic PLLs compared to those without a history of extrathoracic cancer: 25% (13/51) vs. 4.5% (5/109), respectively. In turn, PLLs of metastatic origin decreased the yield by 85%, with an OR of 0.15. Two studies assessed a history of extrathoracic cancer previous to ENB and suspicion of metastatic PLLs as factors decreasing the diagnostic yield of ENB [22, 23]. We believe the reasons for the decrease in profitability are that most metastases of tumours at the lung level are due to hematogenous dissemination and, additionally, to the development of a metastatic niche that provides the adequate microenvironment for the implantation and growth of disseminated tumour cells [60, 61]. Tsuboi et al. [62] documented a significant difference in the bronchoscopy yield of peripheral lung lesions secondary to primary bronchogenic malignancies versus lung metastases, at 76.5% versus 29.1%, respectively. They found that bronchial airway exposure was present in only 5.1% of the metastases < 2 cm in size. Pulmonary metastases follow a hematogenous spread and are surrounded by non-malignant tissue (fibroblasts, neovasculature, inflammatory cells, and extracellular matrix) [60, 61]. Such dissemination patterns compared to those of bronchogenic carcinoma nodules, plus limited endobronchial exposure, might explain the lower yield observed [62, 63]. We believe that this fact is of great importance for ENB and at the level of bronchoscopy as a diagnostic technique in pulmonology. Finally, using the independent variables associated with the diagnostic yield, we generated a model to predict the diagnostic yield of ENB with good discriminating capacity (area under the ROC curve: 0.83). We plan to validate this model in a future prospective study.

We also assessed the diagnostic performance of the tools and techniques used during ENB [14]. Combination of multiple sampling techniques, particularly TBBx and TBNA, positively impacted the diagnostic yield and a diagnostic yield of 69% was reached when the most common individual techniques were used together: suction catheter + TBBx + BAL + CB + TBNA + bronchial washing (Table 5). Chao et al. also noticed a significant yield increase (18%) when TBNA was added to r-EBUS (78.4%) compared to TBBx and bronchial washing without TBNA (60.6%) [64]. In general, TBNA appears to be underutilised [14], even in cases with pleural distance of ≥ 10 mm. This is likely due to technical difficulties manoeuvring the needle in more distal locations and to concerns about a higher pneumothorax risk. However, in our study, its use was not associated with an increased incidence of pneumothorax. Most needle-associated pneumothorax risk have been extrapolated from CT-guided TTNA data (pneumothorax as high as 23%, up to ¼ requiring chest tube drainage) [65]. We believe that TBNA is a safe tool for lesions ≥ 10 mm from the pleura, as seen in our study and recently confirmed in the large multicentre NAVIGATE study [10].

Finally, since our study used stand-alone ENB under moderate sedation, it is possible that the routine use of additional diagnostic tools or general anaesthesia could increase the yield of ENB [14, 22, 29, 33]. For example, Eberhardt et al. reported an 88% yield for combined ENB + r-EBUS versus r-EBUS (69%) or ENB (59%) alone [22]. Our study bears several limitations, including its single-centre, retrospective observational nature, which exposes it to the risk of an unmeasured confounder and might limit the generalisability of the results. Further, our samples were analysed by the same pathologist. This can introduce bias to the diagnostic yield of various sampling techniques once an initial sample is diagnostic. We did not use fluoroscopy, r-EBUS, or ROSE, which could have potentially increased our diagnostic yield; therefore, our results apply mainly to studies not using such techniques.

## Conclusion

Our findings show that ENB, as a stand-alone diagnostic technique using a multimodality sampling method under moderate sedation, has a good diagnostic yield, mainly in the presence of the bronchus sign and the use of TBNA, without increasing the risk of pneumothorax. Notably, we generated a predictive model for ENB diagnostic yield, which should be prospectively validated to provide more clarity regarding the optimal selection of patients undergoing ENB.

### Supplementary Information

**Additional file 1: Supplementary Table 1.** Sampling tools and techniques used during ENB. **Supplementary Table 2. **PET/CT SUV and malignancy diagnosis.** Supplementary Table 3. **Univariate analysis: association between ENB techniques, lesion characteristics and pneumothorax. **Supplementary Table 4. ** Comparison of studies using ENB as stand-alone diagnostic technique. **Supplementary Figure 1.** Diagnostic yield of the different ENB sampling techniques. 

## Data Availability

The datasets generated during and/or analyzed during the current study are available from the corresponding author on reasonable request.
