# Supplementary material for: The effect of combining different sampling tools on the performance of electromagnetic navigational bronchoscopy for the evaluation of peripheral lung lesions and factors associated with its diagnostic yield
Source: BMC Pulm Med. 2023 Nov 8;23:432. doi: 10.1186/s12890-023-02711-1 (PMC10634141; doi:10.1186/s12890-023-02711-1)
Supplement: Supplementary file 1 — Additional file 1: Supplementary Table 1. Sampling tools and techniques used during ENB. Supplementary Table 2. PET/CT SUV and malignancy diagnosis. Supplementary Table 3. Univariate analysis: association between ENB techniques, lesion characteristics and pneumothorax. Supplementary Table 4. Comparison of studies using ENB as stand-alone diagnostic technique. Supplementary Figure 1. Diagnostic yield of the different ENB sampling techniques. [file 12890_2023_2711_MOESM1_ESM.pdf]

Supplementary appendix

**Supplementary table 1.** Sampling tools and techniques used during ENB
1. Suction catheter: an Olympus® catheter PW-2L-1 was advanced to the lesion while performing a continuous suction using a 20 cc syringe.
2. TBBx: at least 4-6 biopsies were obtained using Alton´s alligator cups biopsy forceps, AF-D1812BU.
3. CB: 3 passes were made using an Olympus® BC-202D-3010 brush
4. TBNA: 3 passes were made using a 21G Olympus® NA 401D-1321D needle.
5. BAL: 5 cc of 0.9% normal saline were instilled through the Olympus® catheter PW-2L-1 catheter and were suctioned back using a 10 cc syringe. Material was sent for microbiology (routine bacterial, fungal, and mycobacterial cultures) and cytology analysis.
6. Bronchial washing: sent for similar analysis as BAL.

Notes: ENB, electromagnetic navigational bronchoscopy; TBBx, transbronchial biopsies; CB, cytology brush; TBNA, transbronchial aspiration; BAL, bronchoalveolar lavage.

Supplementary table 2. PET/CT SUV and malignancy diagnosis

	PET/CT Uptake		
Final diagnosis	SUV ≥3	SUV <3	p-value
Malignant	89	11	<0.001
No-malignant	14	15	

Notes: PET/CT, positron emission tomography/computed tomography; SUV, standardized uptake value

Supplementary figure 1. Diagnostic yield of the different ENB sampling techniques

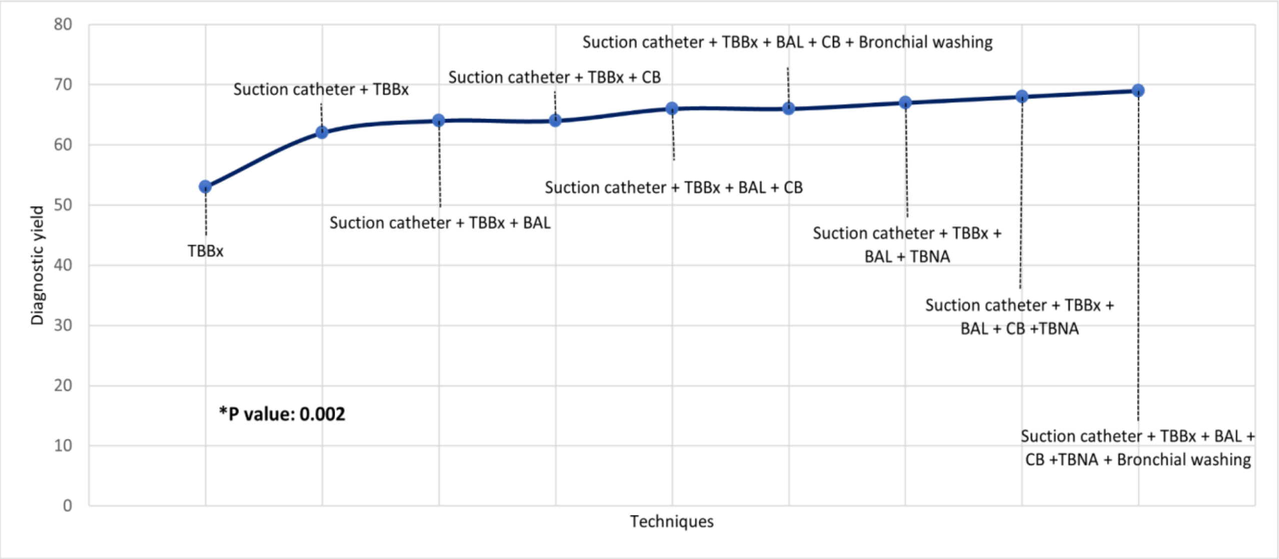

Notes: TBBx, transbronchial biopsies; BAL, bronchoalveolar lavage; CB, cytology brush; TBNA, transbronchial needle aspiration. *trend in the use of transbronchial biopsies and transbronchial needle aspiration with other combined techniques (Cochran–Armitage statistical test)

Supplementary table 3. Univariate analysis: association between ENB techniques, lesion characteristics and pneumothorax

	Pneumothorax		p-value
	Yes	No	
Bronchus sign yes, n(%)	9 (10.3)	79 (89.7)	0.252
Bronchus sign no, n(%)	3 (4.2)	69 (95.8)	
Distance to the pleura ≥10 mm, n (%)	6 (8.5)	64 (91.5)	0.880
Distance to the pleura <10 mm, n(%)	6 (6.6)	84 (93.4)	
Distance to the pleura ≥20 mm, n (%)	3 (6.8)	41 (93.2)	0.840
Distance to the pleura <20 mm, n(%)	9 (7.8)	107 (92.2)	
TBNA yes, n(%)	4 (7.8)	47 (92.2)	1.000
TBNA no, n(%)	8 (7.4)	101 (92.6)	
TBBx yes, n(%)	12 (7.8)	141 (92.2)	0.971
TBBx no, n(%)	0 (0)	7 (100)	
CB yes, n(%)	7 (5.9)	111 (94.1)	0.302
CB no, n(%)	5 (11.9)	37 (88.1)	
Suction catheter + BAL + TBBx +TBB n(%)	4 (6.7)	55 (93.3)	0.383
Suction catheter + BAL + TBBx + TBB+ TBNA n(%)	3 (7.6)	36 (92.4)	
Suction catheter + BAL + TBBx + TBNA n(%)	1 (25.0)	3 (75.0)	
Upper lobes, n(%)	6 (6.4)	87 (93.6)	0.812
Middle lobe, n(%)	1 (6.6)	14 (93.4)	
Lower lobes, n(%)	5 (9.6)	47 (90.4)	
Size < 15 mm, n(%)	8 (12.9)	54 (87.1)	0.061
Size ≥ 15 mm, n (%)	4 (4.1)	94 (95.9)	

Notes: ENB, electromagnetic navigational bronchoscopy; TBBx, transbronchial biopsies; CB, cytology brush; TBNA, transbronchial aspiration; BAL, bronchoalveolar lavage.

Supplementary table 4. Comparison of studies using ENB as stand-alone diagnostic technique

Author, year	Cases	Diagnostic yield	Mean diameter (mm)	Mean follow-up months	General anesthesia	ROSE	Fluoroscopy	r-EBUS
Schwarz [6], 2006	15	69	33.4	ND	No (MS)	No	No	No
Eberhardt [23], 2007 (a)	89	67	28	ND	Yes (61.7%)	No	No	No
Makris [35], 2007	40	62.5	23.5	ND	No (MS)	No	No	No
Bertoletti [45], 2009	53	77.4	31.2	18	No*	No	No	No
Sejio [54], 2010	51	67	25	ND	Yes	No	No	No
Pearlstein [40], 2012	101	82	28	24	Yes	Yes	No	No
Karnak [29], 2013	76	89.5	23.1	24	Yes	Yes	No	No
Loo [33], 2014	40	89.4	26	ND	No (MS)	Yes	No	No
Ozguel [39], 2016	30	71.4	30	6	No (MS)	No	No	No
Raval [46]†, 2016	49	83.3	19.3	24	No (MS)	No	No	No
Yutaka [55], 2021	100‖	74.9	19.4	19	No (LA)	No	No	No
Oh [56], 2021	29	58.6	25.2	ND	No (MS)	No	No	No
Kim [57], 2022	94	81.5	34.3	ND	Yes	No	No	No

Notes: ENB, Electromagnetic navigational bronchoscopy; ROSE, rapid on-site evaluation; r-EBUS, radial endobronchial ultrasound; MS, moderate sedation; LA, local anesthesia; ND, not mentioned

* 50%/50% Nitrous oxide/Oxygen mixture

+A patient by radial endobronchial ultrasound

† Study done using tidal volume expiration-mapped electromagnetic navigational bronchoscopy (Veran Medical Technologies)

‖ 100 lesions: 77 malignant, 23 benign

References

Schwarz Y, Greif J, Becker HD, Ernst A, Mehta A. Real-time electromagnetic navigation bronchoscopy to peripheral lung lesions using overlaid CT images: the first human study. Chest. 2006;129:988-94.

Eberhardt R, Anantham D, Herth F, Feller-Kopman D, Ernst A. Electromagnetic navigation diagnostic bronchoscopy in peripheral lung lesions. Chest. 2007;131 :1800-5.

Makris D, Scherpereel A, Leroy S, Bouchindhomme B, Faivre JB, Remy J, Ramon P, Marquette CH. Electromagnetic navigation diagnostic bronchoscopy for small peripheral lung lesions. The European respiratory journal. 2007;29 :1187-92.

Bertoletti L, Robert A, Cottier M, Chambonniere ML, Vergnon JM. Accuracy and feasibility of electromagnetic navigated bronchoscopy under nitrous oxide sedation for pulmonary peripheral opacities: an outpatient study. Respiration; international review of thoracic diseases. 2009;78:293-300.

Seijo LM, de Torres JP, Lozano MD, Bastarrika G, Alcaide AB, Lacunza MM, Zulueta JJ. Diagnostic yield of electromagnetic navigation bronchoscopy is highly dependent on the presence of a Bronchus sign on CT imaging: results from a prospective study. Chest. 2010;138 :1316-21.

Pearlstein DP, Quinn CC, Burtis CC, Ahn KW, Katch AJ. Electromagnetic navigation bronchoscopy performed by thoracic surgeons: one center's early success. Ann Thorac Surg. 2012;93 :944-9.

Karnak D, Ciledag A, Ceyhan K, Atasoy C, Akyar S, Kayacan O. Rapid on-site evaluation and low registration error enhance the success of electromagnetic navigation bronchoscopy. Ann Thorac Med. 2013;8 :28-32.

Loo FL, Halligan AM, Port JL, Hoda RS. The emerging technique of electromagnetic navigation bronchoscopy-guided fine-needle aspiration of peripheral lung lesions: promising results in 50 lesions. Cancer Cytopathol. 2014;122 :191-9.

Ozgul G, Cetinkaya E, Ozgul MA, Abul Y, Gencoglu A, Kamiloglu E, Gul S, Dincer HE: Efficacy and safety of electromagnetic navigation bronchoscopy with or without radial endobronchial ultrasound for peripheral lung lesions. Endosc Ultrasound 2016;5:189-195.

Raval AA, Amir L: Community hospital experience using electromagnetic navigation bronchoscopy system integrating tidal volume computed tomography mapping. Lung Cancer Manag 2016;5:9-19.

Yutaka Y, Sato T, Isowa M, Murata Y, Tanaka S, Yamada Y, Ohsumi A, Nakajima D, Hamaji M, Menju T, Chen-Yoshikawa TF, Date H. Electromagnetic navigation bronchoscopy versus virtual bronchoscopy navigation for improving the diagnosis of peripheral lung lesions: analysis of the predictors of successful diagnosis. Surg Today. 2022 ;52 :923-930.

Oh JH, Choi CM, Kim S, Jang SJ, Oh SY, Kim MY, Hwang HS, Ji W. Diagnostic Performance of Electromagnetic Navigation Bronchoscopy-Guided Biopsy for Lung Nodules in the Era of Molecular Testing. Diagnostics (Basel). 2021 ;11 :1432.

Kim YW, Kim HJ, Song MJ, Kwon BS, Lim SY, Lee YJ, Park JS, Cho YJ, Yoon HI, Lee JH, Lee CT. Utility and safety of sole electromagnetic navigation bronchoscopy under moderate sedation for lung cancer diagnosis. Transl Lung Cancer Res. 2022 ;11:462-471.
